# Assessment of automated assays for serum amyloid A, haptoglobin (PIT54) and basic biochemistry in broiler breeders experimentally infected with *Escherichia coli*

**DOI:** 10.1186/s13567-022-01040-1

**Published:** 2022-03-21

**Authors:** Sofie Kromann, Rikke Heidemann Olsen, Anders Miki Bojesen, Henrik Elvang Jensen, Ida Thøfner

**Affiliations:** 1 Department of Veterinary and Animal Sciences, Faculty of Health and Medical Sciences, University of Copenhagen, 1870 Frederiksberg, Denmark; 2 DanHatch Denmark A/S, Rugerivej 26, 9760 Vrå, Denmark

**Keywords:** Blood biochemistry, biomarker, acute phase protein, serum amyloid A, haptoglobin

## Abstract

Biomarkers of inflammation are valuable tools for health status evaluation in numerous species. However, in poultry, methods for measuring acute phase proteins (APP) are sparse and rely on manual laboratory labour reserving these parameters mainly for research studies with APP as a focus point. To extend the use of APP beyond tightly focused research studies, blood from experimentally infected and control hens was analysed using equipment available in many veterinary clinics in order to identify easily accessible biomarkers of infection. Blood samples from broiler breeders (*n* = 30) inoculated intratracheally with either *Escherichia coli* or sterile vehicle were randomly selected at 2, 4 and 7 days post-infection (dpi) and subjected to biochemical analysis. Samples for bacteriological testing were collected, and all animals were subjected to a full necropsy for disease confirmation. Significantly higher levels of serum amyloid A were evident in the infected birds at 2 and 4 dpi (*p* < 0.01) compared to the controls. Likewise, haptoglobin (PIT54) levels were significantly elevated at 4 dpi (*p* < 0.01) in the infected animal, whilst at 2 dpi magnesium and calcium were significantly lower in the infected group (*p* < 0.05). Gross pathology and bacteriology confirmed the presence of infection in the *E. coli* inoculated birds. In conclusion, equipment routinely used in other species for rapid analysis of blood samples, successfully differentiated between sick and healthy birds, hereby, showing great potential as an easily added parameter of evaluation in research studies, and as a valuable decision-making tool for poultry veterinarians.

## Introduction

Acute phase proteins (APP) are commonly used as biomarkers of inflammation in humans and the veterinary clinic serving as a diagnostic and prognostic tool [1–4]. Furthermore, APP are also used in order to evaluate response to therapy and in the overall health monitoring of patients [1].

In poultry, the use of AAP is still in its infancy though a number of potential biomarkers have sporadically been investigated in a variety of conditions [5].

Commonly, analysis of APP in poultry relies on methods such as ELISA [5–8] thus reserving these biomarkers almost exclusively to research projects in avian species [5].

Facilitating the use of APP in a broader setting would require rapid and simple methods of analysis, which could provide poultry veterinarians with a diagnostic tool to aid decision-making and, furthermore, enable the establishment of reference intervals linked to birds of different species, breeds, and ages. Additionally, straightforward, and easily available methods for evaluation of APP in birds would prompt their use in experimental studies adding an objective mean for group comparison, as well as aid the evaluation of health status in live animals.

Therefore, the aim of the current study was to identify potential biomarkers of inflammation in broiler breeders infected experimentally with *E. coli*, using standard, rapid and easily accessible veterinary technologies.

## Materials and methods

### Animals and housing

This study was performed utilising material collected from an experimental infection study recently described [9]. Briefly, Ross 308 broiler breeder hens (29 weeks of age) were allocated to groups by randomisation, acclimatised for one week and housed in coops (0.432 m^2^/hen) with wood shavings, enrichment (stray, hay, dust-bath, shelves, and perches), nests and water provided ad libitum. The hens were fed commercial wholefood for egg-laying hens once a day (155/g/hen) with sunflower seeds, wheat- and barley kernels, and fish meal as supplement. A light–dark cycle of 12 h with 30-min dim-phases was maintained and morning temperatures ranged between 19.4 and 26.6 °C within the facilities.

### Inoculum

The inoculum was prepared from *E. coli* ST117 (accession number LXWV00000000.1), originally isolated from a case of colibacillosis and stored at −80 °C, that was streaked onto blood agar base supplemented with 5% bovine blood and incubated overnight at 37 °C. Fifty mL sterile tubes containing 10 mL of Lysogeny broth (LB) were then prepared by adding a colony to each tube. The tubes were then vortex-mixed, and incubated with shaking (125 rpm) at 37 °C for 19 h providing an overnight culture. Thereafter, 250 mL Erlenmeyer glass flasks containing 50 mL of LB were prepared by adding 500 µL of the overnight culture, which was then incubated at 37 °C and shaken (125 rpm) for 4 h, hereby, providing an exponential phase inoculum. Immediately afterwards, the optic density (OD) was measured (OD = 1.3 previously determined equivalent to 1 × 10^9^ CFU/mL at the facilities), whereafter the inoculum was diluted targeting a concentration of 1 × 10^6^ CFU/mL, placed on ice and ten-fold dilutions were made in duplicates and plated onto Lysogeny agar for CFU confirmation. Sterile vehicle (LB) for sham-inoculation of the control group was maintained under conditions similar to the inoculum.

### Inoculation and euthanasia

Syringes with a buttoned steel canula were prepared containing 1 mL of either inoculum or sterile vehicle and placed on ice immediately thereafter. Inoculation was carried out as follows: an assistant fixated each hen briefly while gently opening the beak, enabling a veterinarian to carefully introduce the buttoned canula into the trachea for deposition.

Euthanasia was carried out 2 days post-infection (dpi), 4 dpi and 7 dpi by induction of unconsciousness by blunt force head trauma followed by cervical dislocation. The animals were appointed to each day of euthanasia by randomisation.

### Post-mortem evaluation

The animals were thoroughly necropsied in a blinded and randomised manner as previously described [9]. Briefly, registration of lesions was done systematically, and bacteriological swabs were collected from the trachea, lung, right caudal thoracic airsac, liver, spleen, peritoneum, salpinx (magnum and infundibulum) and bone marrow of the femur.

### Sample collection

Blood samples were obtained prior to inoculation and immediately before euthanasia by withdrawing 4 mL blood from the vena ulnaris utilising a 23G canula and a syringe. Immediately thereafter, blood was divided into sterile, non-coated polypropylene tubes for serum preparation (TPP^®^, Switzerland) and lithium heparin-coated tubes (BD Vacutainer^®^) for plasma. The samples were stored overnight at 6 °C, centrifuged at 3000 *g* for 15 min the next day, whereafter the supernatant (i.e., serum or plasma) was pipetted into freeze tubes and stored at −20 °C until analysis.

### Blood biochemistry analysis

Analyses of serum samples from infected (*n* = 15) and control birds (*n* = 15) were performed at the Veterinary Diagnostic Laboratory at Copenhagen University, Denmark in a randomised and blinded manner utilising the automated analyser Advia 1800 (Siemens, Germany). The following parameters were analysed: total protein content, albumin, alanine aminotransferase (ALAT), alkaline phosphatase (BASP/ALP), total bilirubin, bile acid, aspartate aminotransferase (ASAT), creatinine, blood urea nitrogen (BUN), creatinine kinase, gamma-glutamyltransferase (GGT), calcium, phosphate, magnesium, and SAA. For SAA determination the VET-SAA (Eiken Chemical Co., Tokyo, Japan) based on monoclonal rat anti-human SAA1 antibody, previously successfully applied for SAA analyses in multiple species [10, 11], was used. Plasma samples were analysed for PIT54 levels, analogous to haptoglobin [12], at GD Animal Health (Royal GD Deventer, Netherlands) in a blinded and randomised manner utilising an assay routinely used for haptoglobin measurements in different animal species by this laboratory.

### Ethics

The study was approved by the Danish Animal Experiments Inspectorate under the Danish Ministry of Environment and Food (license no. 2019-15-0201-01611), and all procedures were performed in agreement with this licence, the ARRIVE guidelines and the EU directive 2010/63. The animals were observed every 30 min during the first 6 h after inoculation, and thereafter at 8-h intervals for 3 days following inoculation with an increased frequency if clinical signs occurred. Predefined humane endpoints were determined, and if clinical signs were present, e.g., depression, anorexia, ruffled feathers, lethargy or dyspnoea, the hen was either treated with 0.1 mg/kg buprenorphine and observed with increased frequency or euthanised if refractory to treatment [9].

### Statistics

The statistical analyses were performed using GraphPad Prism version 9.0.1 for Mac OS X (GraphPad Software, Inc., La Jolla, USA). Normality was assessed by the Kolmogorov–Smirnov normality test. In case of parametric data, comparison was made utilising a T-test, whilst non-parametric data were analysed utilising the Mann–Whitney U-test. The data is presented with mean ± SD and the significance level was set at *p* < 0.05. Randomisation was performed using the rand() function Microsoft^®^ Excel for Mac version 16.56.

## Results

### *E. coli* exposure and clinical observations

Animals inoculated with *E. coli* received 7.4 × 10^6^ CFU, whilst the vehicle was confirmed to be sterile. Birds within the infected group developed clinical signs and preterm euthanasia was required due to mild symptoms rendering all birds from this group to be culled by the end of 6 dpi. One bird was euthanised earlier due to symptoms (lethargy and anorexia) refractory to pain relief. Consequently, only four birds remained at 6 dpi resulting in *n* = 14 in the infected group. Preterm euthanasia was primarily due to periodic open mouth breathing and inappetence. Buprenorphine treatment was required for two infected birds surviving to the end of the study, with one bird receiving pain relief at 1 dpi and 2 dpi and the other at 5 dpi. None of the control animals developed clinical signs and all survived to the end of the study.

### Gross pathology

Infection of the inoculated birds was confirmed during necropsy with marked pathological manifestations described previously in detail [9] and summarised briefly in Table 1. On the contrary, only one control bird at 2 dpi presented gross changes potentially related to infection (Table 1).

**Table 1:** **Overview of gross pathology and bacteriology at the time of euthanasia.**

	Infected (*n* = 14^a^)				Control (*n* = 15)			
	Gross pathology		Microbiology		Gross pathology		Microbiology	
	Airways	Systemic	Airways	Systemic	Airways	Systemic	Airways	Systemic
2 dpi	5/5^b^	2/5	5/5	2/5	1/5	0/5	2/5^c^	0/5
4 dpi	3/5	1/5	5/5	3/5	0/5	0/5	3/5^c^	1/5^c^
6-7^d^ dpi	4/4	2/4	3/4	0/4	0/5	0/5	0/5	0/5

dpi: days post-infection, *n* number.

^a^One animal was euthanised preterm due to animal welfare concerns.

^b^At 2 days post-infection (dpi), lesions were evident in the airways of five out of five euthanised animals.

^c^Bacterial growth (sparse) consisting of mixed cultures.

^d^All infected birds were euthanised by the end of 6 dpi due to mild clinical signs.

### Bacteriology

A summary of the results is available in Table 1. All positive samples from the control group consisted of sparse growth of mixed cultures dominated by *E. coli* and originated solely from birds without any lesions, unlike the positive samples from the infected group, which were all from animals with marked lesions of colibacillosis and presented *E. coli* in pure cultures with abundant growth (bacterial lawn or carpet-like).

### Blood biochemistry analysis

**Figure 1:**
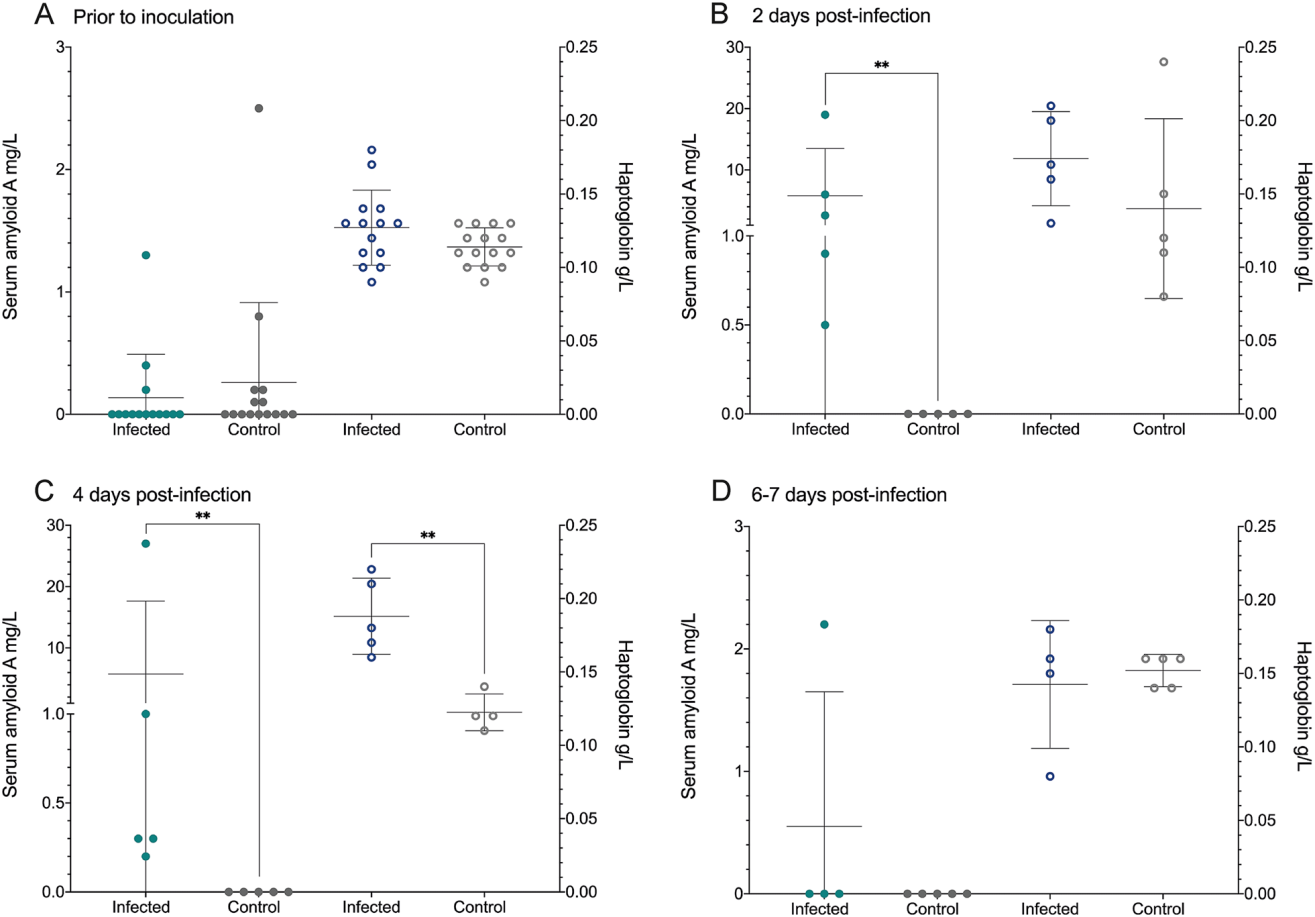
**Serum amyloid A and haptoglobin (PIT54) measurements.** Closed circles = serum amyloid A (SAA). Open circles = haptoglobin (PIT54). Each graph represents different time points. At 4 days post-infection (dpi), it was only possible to collect a sufficient amount of heparin-stabilised blood for haptoglobin (PIT54) analysis from four infected animals. SAA measurements from infected and control animals were compared using a Mann–Whitney test. The haptoglobin (PIT54) data were compared utilising an unpaired t-test prior to inoculation, at 2 and 4 dpi, whilst a Mann–Whitney test was used for comparison of data on 6–7 dpi. **p < 0.01.

Independent of sampling time, the following parameters did not differ between the infected and control animals: total protein content, albumin, ALAT, ALP, bile acid, ASAT, creatine, BUN, GGT and phosphate (Table 2). Throughout the study, a significantly lower level of total bilirubin was present in the infected group compared to the control (*p* < 0.05), and in both groups there seemed to be a steady increase. Creatine kinase fluctuated considerably in both groups but differed between the groups at 7 dpi with the level being higher in the infected group. At 2 dpi, calcium and magnesium was significantly lower in the infected group compared to the control (*p* < 0.05), whereas SAA had risen to a significantly higher level in the infected group (*p* < 0.01). At 4 dpi, this difference in SAA was still present (*p* < 0.01), whilst it was at an equal level at 6–7 dpi (Table 2 and Figure 1). From a single animal in the control group, it was not possible to collect a sufficient amount of blood for a heparin stabilised sample rendering haptoglobin (PIT54) analyses impossible at 4 dpi. Still, a significant difference in haptoglobin (PIT54) levels was evident at this time point (*p* < 0.01) (Table 2 and Figure 1).

**Table 2:** **Overview of blood biochemistry.**

Parameter	Infected (*n* = 14^a^)				Control (*n* = 15)			
	Prior to inoculation	2 dpi	4 dpi	6^b^ dpi	Prior to inoculation	2 dpi	4 dpi	7 dpi
Total protein (g/L)	46.43 ± 3.79	45.95 ± 3.39	48.69 ± 3.37	45.80 ± 2.85	47.38 ± 3.03	49.81 ± 7.16	46.46 ± 1.68	48.82 ± 1.29
Albumin (g/L)	23.04 ± 1.53	22.83 ± 1.25	22.29 ± 1.61	21.94 ± 1.2	23.74 ± 1.75	24.91 ± 3.19	22.4 ± 1.0	23.16 ± 1.0
Alanine aminotransferase (U/L)	2.43 ± 1.65	1.8 ± 0.45	2.0 ± 1.23	2.25 ± 0.96	2.2 ± 1.15	2.6 ± 1.52	2.0 ± 1.0	3.2 ± 0.84
Alkaline phosphatase (U/L)	305.2 ± 69.90	327.8 ± 71.1	322.0 ± 118.4	335.4 ± 118.7	314.9 ± 76.26	321.6 ± 14.33	348.2 ± 60.12	264.6 ± 66.08
Total bilirubin (µmol/L)	3.93 ± 1.49^*^	2.8 ± 1.1^*^	4.8 ± 2.17^*^	5.75 ± 0.96^*^	5.47 ± 2.03	5.8 ± 1.92	7.0 + 1.4	8.6 ± 0.89
Bile acid (µmol/L)	26.71 ± 27.31	43.4 ± 13.94	36.8 ± 12.03	59.5 ± 30.16	14.0 ± 6.56	71.8 ± 36.29	34.6 ± 6.35	56.6 ± 34.59
Aspartate aminotransferase (U/L)	255.9 ± 48.72	307.6 ± 124.1	238.4 ± 55.02	301.5 ± 183.8	275.7 ± 46.33	297.4 ± 59.93	266.8 ± 22.08	227 ± 23.69
Creatinine (µmol/L)	11.5 ± 5.43	14.6 ± 3.05	10.8 ± 1.92	18.0 ± 8.83	8.87 ± 1.96	13.4 ± 4.72	9.6 ± 2.61	8.4 ± 3.36
Blood urea nitrogen (mmol/L)	1.84 ± 2.81	0.94 ± 2.1	2.16 ± 1.96	1.825 ± 3.39	3.02 ± 3.18	5.2 ± 3.33	4.04 ± 2.59	0.0 ± 0.0
Creatine kinase (U/L)	3711 ± 1540	5345 ± 4470	2918 ± 1615	4511 ± 673.3^*^	3895 ± 2117	4224 ± 1646	3234 ± 1905	2954 ± 971.4
Gamma-glutamyl transferase (U/L)	11.64 ± 3.52	14.2 ± 2.39	14.8 ± 6.42	10.0 ± 2.94	12.0 ± 3.89	13.4 ± 4.28	14.0 ± 4.74	13.2 ± 1.74
Calcium (mmol/L)	5.88 ± 0.56	4.82 ± 0.7^*^	5.38 ± 1.18	5.55 ± 1.2	5.86 ± 0.59	6.06 ± 0.57	6.33 ± 0.15	6.27 ± 0.15
Phosphate (mmol/L)	2.32 ± 0.36	2.08 ± 0.26	2.54 ± 0.52	2.55 ± 0.45	2.27 ± 0.35	2.46 ± 0.64	2.3 ± 0.07	2.18 ± 0.36
Magnesium (mmol/L)	1.71 ± 0.21	1.42 ± 0.22^*^	1.6 ± 0.34	1.57 ± 0.31	1.76 ± 0.23	1.93 ± 0.27	1.82 ± 0.7	1.87 ± 0.15
Serum amyloid A (mg/L)	0.14 ± 0.35	5.8 ± 7.7^**^	5.76 ± 11.88^**^	0.55 ± 1.1	0.26 ± 0.65	0.0 ± 0.0	0.0 ± 0.0	0.08 ± 0.18
Haptoglobin (PIT54) (g/L)	0.13 ± 0.03	0.17 ± 0.03	0.19 ± 0.03^**c^	0.14 ± 0.04	0.11 ± 0.013	0.14 ± 0.06	0.12 ± 0.013	0.16 ± 0.02

All comparisons were made between the infected and control at corresponding days utilising an unpaired t-test for analysis of parametric data and a Mann–Whitney test for non-parametric data.

dpi: days post-infection, *n*   number.

^a^Preterm euthanasia due to animal welfare concerns.

^b^All infected birds were euthanised by the end of 6 dpi due to mild clinical signs (inappetence).

^c^It was only possible to collect a sufficient amount of heparin stabilised blood for haptoglobin (PIT54) analysis from four infected birds at 4 days post-infection (dpi).

^*^p < 0.05, ^**^p < 0.01.

## Discussion

By application of automated analyses routinely used in veterinary clinics, a successful differentiation between infected and control animals was obtained, with results showing a significant difference in classic biomarkers of inflammation.

Successful infection of the *E. coli*-receiving birds was confirmed by post-mortem findings of characteristic colibacillosis-lesions and recovery of abundant growth of *E. coli* in pure cultures. A limited number of control animals revealed sparse bacterial growth in mixed cultures. Within the control group, lesions were completely absent upon post-mortem examination, except in a single bird showing mild changes to the trachea at 2 dpi, likely linked to slight mechanical trauma due to the route of inoculation. The absence of inflammatory lesions combined with sparse bacterial growth indicates contamination from the environment during necropsy.

SAA increased considerably in the infected birds acting as a major APP which is in accordance with previous reports on SAA in poultry applying ELISA kits for measurements [6, 7]. In previous studies, quite different levels of SAA in healthy chickens have been reported [6, 7, 13] which might be linked to, e.g., the assay applied, antibody used, different methods of stabilising the blood samples, or it could be linked to factors such as breed or age of the birds. In the current study, SAA levels were mostly undetectable in control animals and seemed to normalise in the infected birds towards the end of the study.

As reported in other species, a “true” gold standard for measurement of APP is lacking [14, 15], thereby prohibiting comparison of results to such an assay. Furthermore, the substantial difference in the reported levels in poultry brings additional challenges to the evaluation of results obtained while measuring APP. However, in the current study, the applied assay is based on monoclonal antibodies for SAA detection, hereby providing an inherent superiority in regard to detection specificity, and importantly, inter-batch consistency [10]. A trait that could also reduce the apparent inter-laboratory variation currently limiting the comparison of results.

When measuring major APP, such as SAA, the diagnostic sensitivity is highly aided by their relative increase from almost undetectable levels in healthy animals to a vast increase in response to inflammation, as seen in the present study.

PIT54, corresponding to haptoglobin in mammals, only differed significantly between infected and control birds at 4 dpi, though a tendency of a higher level in the infected birds was evident at 2 dpi. A rise in this parameter, however, less pronounced than in SAA, agrees with previous reports [6, 12, 16] classifying haptoglobin (PIT54) as a minor APP in birds.

At 2 dpi, magnesium and calcium were significantly lower in the infected animals compared to the controls, which may be due to an uncontrolled balance of ionic concentration across the membranes of damaged cells [17].

Throughout the study, including prior to inoculation, the infected group had a lower level of total bilirubin, which therefore could be coincidental. Yet, bilirubin has previously been described as an antioxidant [18] resulting in increased usage and lowered serum levels due to tissue damage as was evident in the infected birds. Another likely coincidental finding during the study, was the difference in creatinine kinase levels at 6 dpi as this parameter fluctuated considerably throughput the study.

An important aspect of utilising standard equipment and automated analysis, with widespread availability, is the prospect of determining reference intervals in various types of birds, ages, and production stages, such as at the peak of lay in contrast to periods of less physiological stress. Furthermore, the rapid genetic changes occurring in commercial poultry breeds likely demands a continuous adjustment of the intervals, which require the use of quick routine assays.

In conclusion, the present study shows promising potential of standard veterinary equipment for easy evaluation of biomarkers linked to infection in poultry with a future prospect of aiding decision-making for field veterinarians, and adding additional strength to the results in, e.g., experimental infection studies performed in avian species.

## Data Availability

Data, from which the conclusions in this manuscript relies, are presented within the paper.
